# Potentials and challenges of using co-design in health services research in low- and middle-income countries

**DOI:** 10.1186/s41256-023-00290-6

**Published:** 2023-03-13

**Authors:** Devendra Raj Singh, Rajeeb Kumar Sah, Bibha Simkhada, Zoe Darwin

**Affiliations:** School of Human and Health Sciences, University of Huddersfield, Huddersfield, UK

**Keywords:** Co-design, Potentials, Challenges, Health services research, Low-middle-income countries

## Abstract

Co-design with people having poor access to health services and fragile health systems in low- and middle-income countries can be momentous in bringing service users and other stakeholders together to improve the delivery and utilisation of health services. There is ample of evidence from high-income countries regarding how co-design can translate available evidence into developing acceptable, feasible, and adaptable health solutions in different settings. However, there is limited literature on co-design in health research in the context of low- and middle-income countries. Therefore, it is crucial to understand how knowledge about collaborative working can be translated into policy and practice in the context of low- and middle-income countries. Thus, this paper discusses the concept of co-design, co-production, and co-creation in health and the potentiality and challenges of using co-design in health services research in low- and middle-income countries. Despite the challenges, the co-design research has considerable potential to encourage the meaningful engagement of service users and other stakeholders in developing, implementing, and evaluating real-world solutions in low- and middle-income countries. It is essential to balance power dynamics in a co-design process through mutual recognition and respect, participant diversity, and reciprocity and flexibility in sharing. The inclusive and collaborative approach to working is complex due to existing rigid hierarchical structures, socio-cultural beliefs, political interference and working practices. However, this could be minimised by developing transparent terms of reference that reflect the value and benefits of equal partnership in particular co-design work.

## Background

The health systems of low- and middle-income countries (LMICs) are grappling to overcome the inadequacy of human resources, budget insufficiency, poor infrastructure, distrust in government health services, and high inequalities in delivering quality health services [1, 2]. Improving the quality of care and universal accessibility of essential health services is the catchphrase for politicians and bureaucrats in LMICs [3, 4]. However, limited initiatives are implemented to meet the real healthcare needs of the population [4]. The experiences of researchers and practitioners working especially in system-based settings realised that the effective design and implementation of any health innovation requires meaningful collaboration or engagement of researchers, service users, service providers, policymakers, and other concerned stakeholders [5]. This realisation has brought the transference from the traditional thinking about positioning health service users as passive recipients of produced services to meaningful involvement in the service planning, implementation and evaluation process, which has shaped the notion of co-design in the health [6]. Co-design in health and related sectors involves collaboration between researchers, service users, and other stakeholders to define problems and develop, implement and evaluate the solutions in real-world settings [7, 8]. This partnership in conducting research is a democratic process that encourages the involvement of service users from the study's inception (knowledge generation) to the dissemination (translation) of the knowledge and influencing the strategies [9]. This concept of collaborative work broadly fits under the discipline of implementation research (IR), where understanding the “context” and “actors” are critical to the success of any interventions [10]. Understanding specific interventions, such as “what works in what context” and “why and how it works in a specific context”, is crucial for the success of any intervention implementation, its scalability, and sustainability [10]. The concept of co-design has been diversely defined by authors and practitioners of various disciplines considering its applications in different contexts [8, 11]. However, the fundamental understanding of co-design is embedded in the principle of creatively engaging service users (end-users) and other stakeholders in assessing complex problems and developing pragmatic solutions collectively [8]. Stakeholders in this context more precisely refer to service users, community representatives, civil societies, and different levels of government and non-government entities who have concerns and understanding of the local environment and resources [12].

The history of collective, collaborative, or participatory design can be traced back to the 1970s in western society when people started to practice joint efforts to achieve their organisational and societal goals [13]. Today, the concept of the co-design process closely reflects the essence of the traditional participatory action research method [14]. However, the co-design is more than the participatory process [8]. Participatory action research is a reflective enquiry process that encourages recipients’ (service users and stakeholders) involvement in cooperating with designers, researchers and developers during innovation to generate actional knowledge [15]. On the other hand, co-design is a collaborative process that favours enabling the recipients to produce practical outcomes which are beyond the actional knowledge [7, 8], for example, from developing a realistic joint plan to implementing and evaluating it or from agreeing to re-distribute the budgets to genuinely redistributing them.

Co-design in high-income countries is considered an invaluable way of engaging the stakeholders while developing a service, policy, or other interventions [16, 17]. Previous literature shows that not all kinds of participation in the participatory process refer to genuine participation according to the ladder concept of the citizen participation [18]. Therefore, practical approaches are essential for the meaningful engagement of stakeholders to have authentic contributions. Further, literature shows that co-design in high-income countries (HICs) has been best practiced for involving indigenous, vulnerable and marginalised communities in the research process to develop user-centred services [19, 20]. For example, experience-based co-design was found suitable for improving health services in different areas, including mental health, adolescent health, geriatric health, maternal health etc., in the UK, USA, Australia, Canada and other HICs. This is because the design process involves users or those affected by the co-designed product, such as service providers, policymakers, etc. [21, 22]. However, co-design practice in resource-poor countries is still at its inception stage with limited understanding and applications in the health research [23]. The inadequacy of appropriate co-design skills, scarcity of resources, lack of trust with stakeholders and challenges to bringing marginalise communities into the design process due to their experience of previous false assurance and exploitations in the name of community development works by different agencies are also limiting the application of co-design in these settings [22, 23]. Moreover, different additional factors influence the co-design process, such as cultural, economic, environmental, political, and other structural factors [24, 25]. So, it is imperative to have insight into how this differentiation of contexts is expected to enable or impede co-design in health services research in LMICs. Furthermore, there needs to be more evidence and discussion about how co-design research can benefit the health system of LMICs. Therefore, this paper aims to discuss the concept of co-design, including related concepts of co-production and co-creation, and explore its potentials and challenges in health services research within the context of LMICs. The co-design-related literature published between 2000 and 2022 was considered as the foundation for this perspective piece of work.

## “Co-design” or “co-production” or “co-creation”

“Co-design”, “Co-production”, and “Co-creation” are the most recent forms of participatory and collaborative approaches in health research. It has often been used interchangeably [11], despite the conceptual differentiations [8, 11]. The key feature that all these concepts share is the emphasis on the genuine participation and inputs from service users and stakeholders in the design, delivery and evaluation of the services, initiatives or innovations.

Co-design is defined as “a process of collaborative design thinking or a joint inquiry and imagination where different participants associated with the design process work together to identify the problem, develop solutions, and evaluate those solutions” [7]. The Design Council of the United Kingdom defined ‘co-design’ as *“the meaningful involvement of end-users in the design process”* [26]. Co-design can be either future-focused (prospective planning) or concurrent (parallel planning within the existing context) [27]. The key idea of co-design is that service users are viewed as ‘experts’ of their own experiences and are central to the design process. Therefore, co-design is an active collaboration process involving different people with specific knowledge and experiences, providing an equal level of power to be creative and innovative to produce outputs such as health policy, practice manuals, strategies, new services, initiatives, etc. [11]. In addition, the concept of co-design also embraces and promotes one of the core principles of the Declaration of Alma-Ata on primary health care, where community participation was recognized as essential to the primary health care [28].

Co-production is the process where inputs from service users and other stakeholders are used to produce, deliver and commission a feasible service for the public [29]. The key consideration is that co-production is a vital phase in service delivery that usually comes after the co-design phase [30]. Co-production is a long-term relationship between professionals and service users where power, information, and decision are shared to achieve expected outcomes. Also, co-production is viewed as an implementation process of previously determined solutions to previously agreed problems with an emphasis on the redistribution of the existing resources for achieving the optimum outcomes of the interventions or initiatives [8]. Therefore, the successful delivery of the agreed interventions could only be achieved with sufficient inputs and active collaboration of service users, implementers and other stakeholders.

Co-creation embraces the collaboration of the service users and other stakeholders at all stages of the creative problem-solving process, from design, production, and implementation to evaluation of the solution [8]. Local initiators set the plan for collaboration among the stakeholders, for example, in the health sector, local health agencies, local government, community representatives, service user representatives, civil society organisations and others [8, 31]. Co-creation in a resource-constrained setting has been defined as *“iterative interaction that empowers resource-constrained communities and integrates their knowledge and capabilities with those of a company and other actors throughout the process of designing solutions”* [32]*.* Co-creation refers to bringing something together into existence i.e., adding value to a product or service in a collaborative way [27, 33]. For example, the services do not have their inherent value unless the value is created within the context. The creation of value to services or products is co-created by transforming the core components of the product or service while co-producing the services or product [33]. In simple terms, co-creation is a process to jointly co-create value-based services that best suit the context of service users [8]. The similarities and differences between co-design, co-production and co-creation can be compared from different aspects such as approaches in stakeholder engagements, principles, intended outcomes, creative levels, etc. (Table 1).

**Table 1:** Differences and similarities between co-design, co-production, and co-creation

Characteristics	Co-design	Co-production	Co-creation
Stakeholder involvement	Service users have opportunities to be involved in the beginning for identifying the problem, designing solutions, and modifying the solutions after prototyping or selecting the best from the list of alternatives. Active, equal and reciprocal relationship	Service users’ involvement is relatively passive and organizational or expert-centric. Passive role and rely on contextual setting and resources	User-centric and experience-centric or likely to be based on experience Very active and provide continuous inputs to service providers for value creation
Common methods of stakeholder engagements	Social learning, network mapping, journey mapping, reflexive practice, interviews, group discussion, workshop, survey, nominal group technique, etc.	Interviews, group discussion, workshop, patient and public involvement, Delphi technique, nominal group technique, etc.	Interviews, group discussion, workshop, patient and public involvement, social learning, etc.
Key principles	Inclusiveness, genuine participation, development-oriented, ownership and power sharing, responsiveness, iterative process, outcome-focused	Equality, diversity, building on people’s capabilities; reciprocal relationships; shared cultured	Inspire to participate; trust on process, lead the change, people first, shared results, connect creativity, pertinent partners, continued development
Intended outputs	Identify the problem, develop, and prototype solutions	Implement the co-design/proposed solutions	Design, implement and evaluate the solution, and value creation of the co-designed product or service
Creative levels	Planning, development and designing	Production and scalability	Adopting and using or consumption

## The importance of ‘context’ in co-design

**Fig. 1:**
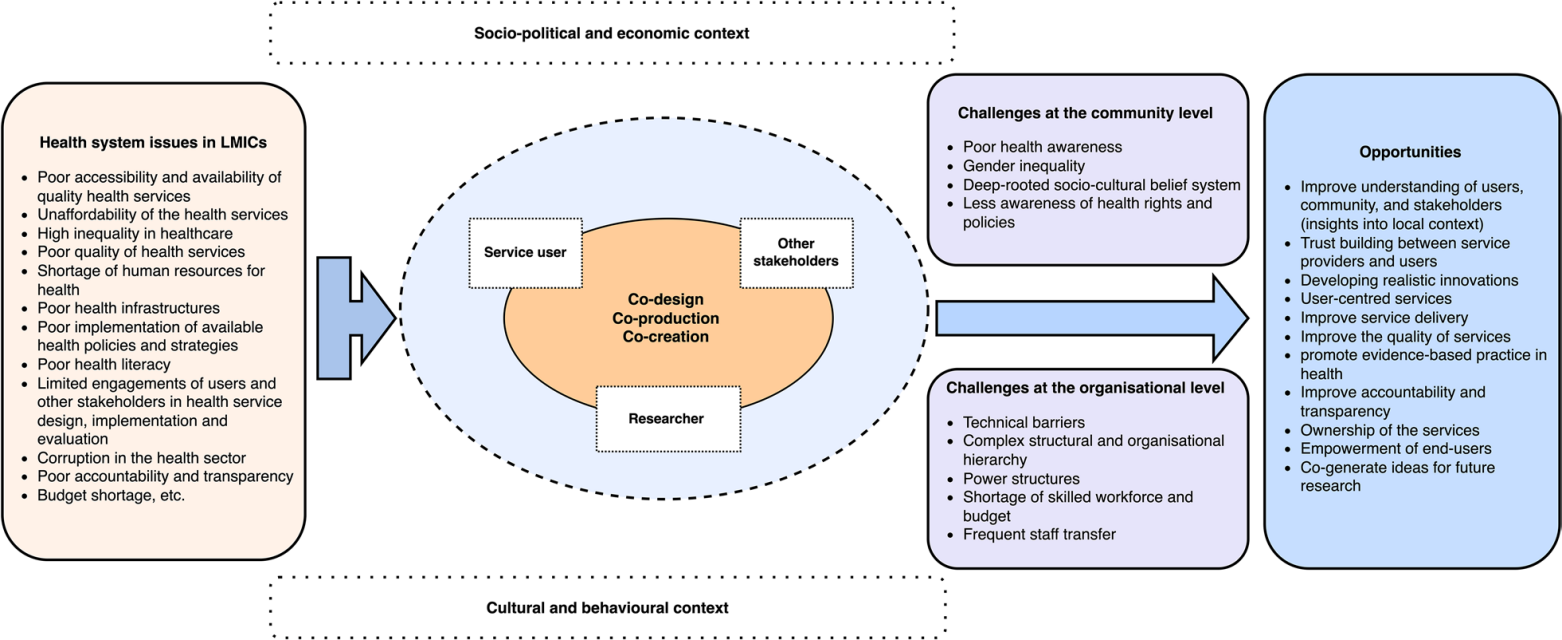
Challenges and opportunities of co-design in the health services of LMICs, with consideration of context

The development of real-world solutions demands the understanding and acknowledgement of the complexity of the problems [34]. Most health problems are primarily influenced by a wide range of interlinked contextual factors of a particular society or nation, such as social, political, economic, cultural, behavioural, etc. [22, 34, 35]. For example, the determinants of maternal and newborn health in one socio-cultural context differ from the other. Therefore, it should not be assumed that solutions co-designed in resource-rich settings can be imported into resource-poor settings. Solutions must be context-informed, necessitating co-design to be attempted in resource-poor settings [34]. Recognising the critical roles of context in a co-design process could create a smooth pathway toward addressing complex health issues and making sustainable impacts (Fig. 1).

Similarly, it is crucial to pick an appropriate technique to identify key actors, service users, and influencers to be included in the co-design process. Ensuring an adequate understanding of stakeholders’ power levels, positions, and interests are essential components of the co-design process [35, 36]. The unfair and unequal power distributions among different actors in the LMICs context contribute towards creating socioeconomic and health inequalities [37, 38]. The power of individuals is embedded into the social structure and cultural contexts, but their actions are not determined [36]. The power in the working partnership approach can be reflected as observable conflicts or hidden omissions of possible alternatives where choices and agendas can favour more power holders [39]. These power holders in the LMICs context could be funders, local authorities, political leaders, government staff, health professionals, intellectuals, etc. [31, 40]. Also, the participant’s gender, illiteracy, language, and socio-economic and ethnic marginalisation can make them feel powerless and hamper their engagements in the co-design process [25, 40]. Thus, it is essential to balance power dynamics in a co-design process through mutual recognition and respect, diversity in the participants, use of common language, independence and sense of security to participate equally, reciprocity and flexibility in sharing; all of this should apply from the pre-commencement stage of co-design [36, 41]. Likewise, transparent dialogue among stakeholders can support creating space for adjustments to fit all levels of participants into a common framework and avoid deliberation that could exclude marginalised participants [36]. Furthermore, it is equally essential to develop a trustful working environment enabling the collaboration process at interpersonal, operational, and system levels for the best possible co-design outcomes [36].

## Challenges for co-design research

Co-design has become a buzzword among researchers and government actors in different sectors. However, it is neither a panacea nor free from challenges when attempting it in a real-world settings [21, 23, 42]. There could be several difficulties associated with embracing co-design principles, processes, and tools when conducted in resource-constrained settings [22]. The key focus in co-design is to design the service for the majority of the service users [7]. However, there is also a need for more clarity and guidelines for bringing those who are in exclusion or face multiple forms of marginalisation, particularly in the LMICs [40, 43]. Nonetheless, co-design is a process intended for innovation to change something in a specific context (sociocultural, economic, political, etc.) [40]. However, this process may take more costs and a longer time than expected to observe the desired outcomes [42], may pose a reluctance to engage participants or discourage the researchers or co-design team from continuing their work [22]. Thus, adequate time and resources are essential to understand the realities, build a trustworthy collaboration and monitor the progress of agreed solutions [22]. Moreover, transparent dissemination about the benefits of service users' inputs, flexible financial and administrative support for the participation and appropriate location of the events can encourage marginalised participants for their meaningful participation in co-design [44]. Therefore, the selection of proper tools and techniques for co-design can be perplexing due to limited knowledge of the applicability and replicability of such tools in LMICs.

The inclusive and collaborative approach to working in LMICs is complex due to existing rigid hierarchical structures, traditional beliefs, political interference and working practices and culture that hinder more equitable collaboration [32, 42]. At the same time, co-design is often contextual, and there is limited evidence to support the replication of the process and convince about the long-term outcomes/impacts of the co-design among users and stakeholders [24, 32, 42]. The poorly interlinked decentralised health system, where different tier health governance and health services delivery structures in the LMICs have multiple priorities, poses structural barriers in co-design [25, 45]. In addition, gender norms and traditional values, futile power politics, poor governance arrangements, reluctance in power-sharing among leaders and bureaucrats, and ambiguity in authority devolution are some institutionalised key challenges for delayed and unaccountable decisions in most of the LMICs’ [25, 45]. These circumstances can challenge the principle of power equalisation in the co-design process. However, this could be minimised by developing transparent terms of reference that reflect the value and benefits of equal partnership in particular co-design work [44]. Also, regular dialogue and recognising and respecting each other knowledge and capabilities can help with critical enquiry and the cultivation of healthy ideas through co-design research.

## Opportunities of using co-design in health services research

The previous studies from developing countries using co-design in health research have explicitly illustrated the potentiality of the user's engagement in the service design, implementation, and evaluation [23, 25]. The practitioners and researchers have suggested the replication and applicability of the co-design methods in health services research in resource-poor countries [23]. The application of this concept is believed to make service users mindful in the service designing process and optimally utilise the available resources [23, 42]. Furthermore, stakeholder engagements via the co-design process help to induce local decision-makers to be more transparent and accountable to the realities, strengths, and constraints of the local context while developing evidence-based health interventions and policies. This kind of stakeholder engagement itself is a communal approach that can potentially reduce several conflicts and discrepancies that mainly arise among different actors within and beyond the health system at local levels of health structures in the LMICs [31]. The participatory approach in co-design involves working together with participants without favouring one type of knowledge over another and conducting the research together for joint ownership [8]. This process can further help to build trust between the service providers and service users, thereby improving the quality and utilisation of health services [22]. The transparent power shifting in the co-design process will also help service users boost their self-confidence and encourage them towards service utilisation and taking control of their health [5]. Moreover, the co-design approach is expected to provide new methodological insight and encourage health services researchers to understand the local context and apply innovative methods in generating evidence-based solutions in resource-constrained settings [42]. The use of co-design with a better understanding of context and background scientific knowledge enhances the support in designing evidence-based solutions (Fig. 1) [23]. However, adequate evidence from the evaluation of co-design work is essential to make it feasible, acceptable, and sustainable for strengthening and maximising the outcomes of the healthcare system within the given context [38].

In addition, the early phase of the co-design process often gathers evidence through interviews or other methods to enhance the co-design process. However, there needs to be more practice in utilising the structural methods informed by the theories of implementation science in the co-design process. Thus, blending implementation science frameworks within co-design can provide a novel approach to developing more realistic forms of co-design work and its successful implementation [46]. There are different types of theories and models used in implementation science. However, the Determinant Framework, Consolidated Framework for Implementation Research, Theoretical Domains Framework and Integrated-Promoting Action on Research Implementation in Health Services is the most widely used frameworks in the implementation science [46]. These frameworks can be used in co-design research to gather live experiences and understand the contexts that can be identified as enablers or barriers at an early design stage that influence the implementation outcomes [46]. It is also argued that evaluating the success and failures of health policies in LMICs is mainly determined by the policy contents and underestimates the roles of stakeholders, policy context, and the policy implementation process [47]. The co-design approach in integration with implementation science can play a vital role in addressing such gaps in engaging the users and other stakeholders at different stages of policy formulation, implementation, and evaluation [5].

## Conclusion

It can be concluded that co-design research has considerable potential to encourage service users and other diverse stakeholders to meaningfully participate in problem identification, service design, implementation and evaluation of the agreed solutions in LMICs. The appropriate use of co-design can support bringing unheard voices of marginalised service users and other stakeholders to strengthen fragile health systems. However, this requires a supportive environment which recognises the importance of multi-stakeholder engagement in collaborative work, building trust among users and other stakeholders, respecting each other capabilities and adopting a transparent approach in co-design work. Critical challenges for conducting co-design research in LMICs could include limited budget and time to demonstrate the intervention outcomes, low literacy, power equalisation, power politics, poor governance, diversity in participation, gender norms, and traditional values. So, we must recognise the contextual differences when compared to other countries where co-design research is more established. Without adequate consideration of the ‘context’, there is potential to limit not only the transferability of the learnings (findings) but also the use of co-design methods. Aspects relating to power imbalance due to complex hierarchal structures need to be actively considered and practical strategies are essential to overcome such barriers in the co-design process. In addition, the successful example of co-design, co-production, and co-creation work in resource-constrained settings should be extensively tested to generate adequate contextual evidence to build an evidence-based practice. Without pursuing co-design in low and middle-income countries, we risk further widening of research inequalities in health services research, and the progress towards better health and wellbeing in the low- and middle-income countries will remain limited.

## Data Availability

Not applicable.

## Abbreviations

HICs: High-income countries
IR: Implementation research
LMICs: Low- and middle-income countries
SDG: Sustainable Development Goal
WHO: World Health Organization

## References

[1] Mills A (2014). Health care systems in low- and middle-income countries. N Engl J Med.

[2] Szabo S, Nove A, Matthews Z, Bajracharya A, Dhillon I, Singh DR (2020). Health workforce demography: a framework to improve understanding of the health workforce and support achievement of the Sustainable Development Goals. BMC Hum Resour Heal.

[3] Khan MS, Hashmani FN (2018). Political and technical barriers to improving quality of health care. Lancet.

[4] Han W (2012). Health care system reforms in developing countries. J Public Health Res.

[5] Batalden M, Batalden P, Margolis P, Seid M, Armstrong G, Opipari-Arrigan L (2016). Coproduction of healthcare service. BMJ Qual Saf.

[6] Bate P, Robert G (2006). Experience-based design: from redesigning the system around the patient to co-designing services with the patient. Qual Saf Health Care.

[7] Steen M. Co-design as a process of joint inquiry and imagination. Des Issues. 2013;29. http://direct.mit.edu/desi/article-pdf/29/2/16/1715163/desi_a_00207.pdf. Accessed 20 Dec 2021.

[8] Vargas C, Whelan J, Brimblecombe J, Allender S (2022). Co-creation, co-design and co-production for public health: a perspective on definitions and distinctions article history. Perspectives (Montclair).

[9] Grindell C, Coates E, Croot L, O’Cathain A (2022). The use of co-production, co-design and co-creation to mobilise knowledge in the management of health conditions: a systematic review. BMC Heal Serv Res.

[10] Peters DH, Adam T, Alonge O, Agyepong IA, Tran N (2013). Implementation research: what it is and how to do it. BMJ.

[11] Brandsen T, Steen T, Verschuere B (2018). Co-production and co-creation: engaging citizens in public services.

[12] Hyder A, Syed S, Puvanachandra P, Bloom G, Sundaram S, Mahmood S (2010). Stakeholder analysis for health research: case studies from low- and middle-income countries. Public Health.

[13] Sanders EB-N, Stappers PJ (2008). Co-creation and the new landscapes of design. CoDesign.

[14] Jagosh J, MacAulay AC, Pluye P, Salsberg J, Bush PL, Henderson J (2012). Uncovering the benefits of participatory research: implications of a realist review for health research and practice. Milbank Q.

[15] Baum F, MacDougall C, Smith D (2006). Participatory action research. J Epidemiol Community Health.

[16] Donetto S, Pierri P, Tsianakas V, Robert G (2015). Experiencebased co-design and healthcare improvement: realizing participatory design in the public sector. Des J.

[17] Bovaird T (2007). Beyond engagement and participation: user and community coproduction of public services. Public Adm Rev.

[18] Arnstein SR (1969). A ladder of citizen participation. J Am Plan Assoc.

[19] Mulvale G, Moll S, Miatello A, Robert G, Larkin M, Palmer VJ (2019). Codesigning health and other public services with vulnerable and disadvantaged populations: insights from an international collaboration. Heal Expect.

[20] Moll S, Wyndham-West M, Mulvale G, Park S, Buettgen A, Phoenix M (2020). Are you really doing ‘codesign’? Critical reflections when working with vulnerable populations. BMJ Open.

[21] Farrington CJT (2016). Co-designing healthcare systems: between transformation and tokenism. J R Soc Med.

[22] Jagtap S (2022). Codesign in resource-limited societies: theoretical perspectives, inputs, outputs and influencing factors. Res Eng Des.

[23] Slattery P, Saeri AK, Bragge P (2020). Research co-design in health: a rapid overview of reviews. Heal Res Policy Syst.

[24] Aranda-Jan CB, Jagtap S, Moultrie J. Towards a framework for holistic contextual design for low-resource settings. Int J Des. 2016;10. http://www.ijdesign.org/index.php/IJDesign/article/view/2596/751. Accessed 18 Feb 2022.

[25] Yadav UN, Lloyd J, Baral KP, Bhatta N, Mehta S, Harris MF (2021). Using a co-design process to develop an integrated model of care for delivering self-management intervention to multi-morbid COPD people in rural Nepal. Heal Res Policy Syst.

[26] Design Council. Eleven lessons: a study of the design process. London; 2005. www.designcouncil.org.uk. Accessed 27 Dec 2021.

[27] Nabatchi T, Sancino A, Sicilia M (2017). Varieties of participation in public services: the who, when, and what of coproduction. Public Adm Rev.

[28] WHO. Declaration of Alma-Ata International Conference on Primary Health Care, Alma-Ata, USSR, 6–12 September 1978. Geneva, Switzerland; 1978. https://cdn.who.int/media/docs/default-source/documents/almaata-declaration-en.pdf?sfvrsn=7b3c2167_2. Accessed 1 Feb 2022.

[29] Ostrom E (1996). Crossing the great divide: coproduction, synergy, and development. World Dev.

[30] Vennik FD, van de Bovenkamp HM, Putters K, Grit KJ (2015). Co-production in healthcare: rhetoric and practice. Int Rev Adm Sci.

[31] Cook N, Siddiqi N, Twiddy M, Kenyon R (2019). Patient and public involvement in health research in low and middle-income countries: a systematic review. BMJ Open.

[32] Nahi T (2016). Cocreation at the base of the pyramid: reviewing and organizing the diverse conceptualizations. Org Environ.

[33] Osborne SP, Radnor Z, Strokosch K (2016). Co-production and the co-creation of value in public services: a suitable case for treatment?. Public Manag Rev.

[34] Schwoerer K, Keppeler F, Mussagulova A, Puello S. Co-designing a more context-based, pluralistic, and participatory future for public administration. Public Adm. 2022.

[35] Litchfield I, Bentham L, Hill A, McManus RJ, Lilford R, Greenfield S (2018). The impact of status and social context on health service co-design: an example from a collaborative improvement initiative in UK primary care. BMC Med Res Methodol.

[36] Farr M (2017). Power dynamics and collaborative mechanisms in co-production and co-design processes. Crit Soc Policy.

[37] Guerra G, Borde E, De Snyder VNS (2016). Measuring health inequities in low and middle income countries for the development of observatories on inequities and social determinants of health. Int J Equity Health.

[38] Yadav UN, Lloyd J, Baral KP, Bhatta N, Mehata S, Harris M (2021). Evaluating the feasibility and acceptability of a co-design approach to developing an integrated model of care for people with multi-morbid COPD in rural Nepal: a qualitative study. BMJ Open.

[39] Lukes S (2005). Power: a radical view.

[40] Jagtap S (2021). Co-design with marginalised people: designers’ perceptions of barriers and enablers. CoDesign.

[41] Ärleskog C, Vackerberg N, Andersson AC (2021). Balancing power in co-production: introducing a reflection model. Humanit Soc Sci Commun..

[42] Jagtap S (2022). Codesign in resource-limited societies: theoretical perspectives, inputs, outputs and influencing factors. Res Eng Des.

[43] Ní Shé É, Cassidy J, Davies C, De Brún A, Donnelly S, Dorris E (2020). Minding the gap: identifying values to enable public and patient involvement at the pre-commencement stage of research projects. Res Involv Engagem.

[44] O’Donnell D, Ní Shé É, McCarthy M, Thornton S, Doran T, Smith F (2019). Enabling public, patient and practitioner involvement in co-designing frailty pathways in the acute care setting. BMC Health Serv Res.

[45] Singh DR, Sunuwar DR, Shah SK, Karki K, Sah LK, Adhikari B (2021). Impact of COVID-19 on health services utilization in Province-2 of Nepal: a qualitative study among community members and stakeholders. BMC Health Serv Res.

[46] Nilsen P (2015). Making sense of implementation theories, models and frameworks. Implement Sci.

[47] Walt G, Shiffman J, Schneider H, Murray SF, Brugha R, Gilson L (2008). ‘Doing’ health policy analysis: methodological and conceptual reflections and challenges. Health Policy Plan.

